# Genetic background and microbiome drive susceptibility to epicutaneous sensitization and food allergy in adjuvant-free mouse model

**DOI:** 10.3389/fimmu.2024.1509691

**Published:** 2025-01-29

**Authors:** Tereza Hornikova, Anna Jelinkova, Zuzana Jiraskova Zakostelska, Tomas Thon, Stepan Coufal, Andrea Polouckova, Eliska Kopelentova, Miloslav Kverka, Peter Makovicky, Helena Tlaskalova-Hogenova, Anna Sediva, Martin Schwarzer, Dagmar Srutkova

**Affiliations:** ^1^ Laboratory of Gnotobiology, Institute of Microbiology of the Czech Academy of Sciences, Novy Hradek, Czechia; ^2^ Laboratory of Cellular and Molecular Immunology, Institute of Microbiology of the Czech Academy of Sciences, Prague, Czechia; ^3^ Department of Immunology, 2nd Faculty of Medicine, Charles University and Motol University Hospital, Prague, Czechia; ^4^ Department of Histology and Embryology, Faculty of Medicine, University of Ostrava, Ostrava, Czechia

**Keywords:** epicutaneous sensitization, food allergy, mast cells, germ-free, microbiome, mouse model of allergy

## Abstract

**Background:**

The dual allergen exposure hypothesis states that sensitization to food antigens occurs through a damaged skin barrier in individuals with no previous oral tolerance to certain foods. However, the resulting allergic reaction could depend on factors such as the host’s genetic predisposition as well as the skin and gut microbiota.

**Methods:**

Specific-pathogen-free BALB/c and C57BL/6 and germ-free (GF) BALB/c mice were epicutaneously sensitized with ovalbumin (OVA) via dorsal tape-stripped skin and challenged with OVA by intragastric gavage. The development of food allergy (FA) symptoms, the Th2 and mast cell immune response and differences in the skin and gut microbiota were investigated.

**Results:**

BALB/c mice, but not C57BL/6 mice, showed severe clinical signs of FA (hypothermia, diarrhea) as well as a stronger serum antibody response and Th2 cytokine secretion in the spleen and jejunum after OVA-treatment. The increased mast cell count correlated with higher MCPT-1 production and histidine decarboxylase mRNA expression in the jejunum of these mice. The 16S rRNA sequencing analysis revealed lower abundance of short-chain fatty acids producing bacteria in the gut microbiome of OVA-treated BALB/c mice. Changes in the β-diversity of the gut microbiome reflect both the genetic background as well as the OVA treatment of experimental mice. Compared to SPF mice, GF mice developed more severe anaphylactic hypothermia but no diarrhea, although they had a higher mast cell count, increased MCPT-1 production in the jejunum and serum, and increased arachidonate 5-lipoxygenase mRNA expression.

**Conclusions:**

We show that the BALB/c mice are a mouse strain of choice for model of adjuvant-free epicutaneous sensitization through the disrupted skin barrier and following food allergy development. Our results highlight the significant influence of genetic background and microbiota on food allergy susceptibility, emphasizing the complex interplay between these factors in the allergic response.

## Introduction

The incidence of food allergy (FA) has increased in recent decades and poses a significant challenge to the health and overall well-being of both pediatric and adult patients. The prevalence of FA varies depending on the diagnostic method, number and type of allergens, and geographic location (1–3). The development of FA is a complex process influenced by genes, host immune responses, epithelial barrier function, and environmental factors. Ultra-hygienic lifestyle, overuse of antibiotic, non-vaginal births and other factors lead to altered microbial communities on the skin and in the gut of infants which are associated with abnormal maturation of the immune system and development of atopy later in life (4). Atopic dermatitis (AD)/eczema with higher disease severity and early onset is a common comorbidity that accompanies the development of food allergies (5). Recently, it has been proposed that a damaged skin barrier due to eczema may allow penetration of food antigens through the skin. In the absence of prior oral tolerance to certain foods, skin exposure leads to food sensitization and allergy in predisposed individuals (6–9). This phenomenon is also known as the dual-allergen exposure hypothesis, which states that early life allergen exposure via the skin could lead to a deviation of T-cells towards a pro-allergic Th2 type and subsequent development of FA. In contrast, early oral exposure to food antigens induces tolerogenic response with Treg cell subtypes (10). In addition, there is increasing evidence for the existence of a skin-gut axis in which inflammation in the skin causes remodeling of the epithelial and immune profile of the intestine in a manner that could promote pathological type 2-biased immune responses and the development of FA (11).

The microbiota of the skin and gut plays an essential role in the recruitment, accumulation and function of various immune cells at mucosal sites, such as T-cells or mast cells (12, 13). It has been shown that skin commensals are essential for functionality of the resident T-cells, strengthening of the skin barrier and education of the keratinocytes to effectively combat skin pathogens. Dysbiosis is an imbalance in the composition and function of the microbiota that disrupts homeostasis and contributes to diseases development (14). In patients with atopic dermatitis (AD) shifts in the skin microbial communities have been well documented, including decreased α-diversity and increased colonization with pathogen *Staphylococcus aureus* (15). The disrupted barrier at inflamed sites of AD lesions likely allows for the translocation of the commensal antigen to the dermis, the development of commensal-specific T-cells and plasma cells, and subsequently promotes the production of bacteria-specific antibodies. Consequently, enhanced microbial exposure on the inflamed skin of AD patients may favor dysregulated antibody-mediated immune selection of bacteria in the gut, contributing to the development of gut bacteria dysbiosis and FA susceptibility (11).

Mouse models are an important tool to better understand the mechanism of food allergy and to explore new treatment options (16, 17). Various methods have been developed for sensitization to food allergens, including intraperitoneal, intragastric, intranasal, subcutaneous and epicutaneous administration (18). Most of these methods require an artificial inflammatory stimulus such as aluminum-containing adjuvants, calcipotriol, bacterial toxins, or the use of a genetically engineered mice to promote a type 2-biased immune response and IgE production. In the epicutaneous adjuvant-free FA mouse model, which also serves as an atopic dermatitis mouse model, simple disruption of the skin barrier via tape-stripping, mechanical raking or prolonged moist bandaging followed by antigen exposure is sufficient to trigger FA sensitization (19). The proposed mechanism is that damage to the skin (due to mechanical injury from scratching, chemical irritants, microbial by-products, or immune system dysregulation) initiate a type 2 immune response in the skin, promoting the expansion and activation of mast cells in the gut, increasing gut permeability and thus facilitating the development of food anaphylaxis (20, 21).

Studies using mouse models have demonstrated that the genetic background of different mouse strains can have a significant and specific impact on the manifestation of allergic diseases in addition to the pathway of sensitization and allergen exposure (22–24). Previously, it has been described that the C3H/HeJ mouse strain mounts robust specific IgE response to peanut sensitization with or without adjuvant with mast cell degranulation, plasma histamine release and anaphylactic reaction upon subsequent allergen exposure (25, 26). The C57BL/6 (B6) mouse strain is the most commonly used strain in biomedical research. The advantage of C57BL/6 over other inbred strains is that most knockout and transgenic models are based on this genetic mouse background (27), which allows the study of the molecular mechanisms of allergy development. In contrast, fifty-nine representative food allergy models have been reported in allergy research, with the BALB/c mouse strain being the most frequently used. However, the results of published research using both mouse strains vary, indicating that different mouse strains must be selected depending on the research purpose, use of adjuvants, route and dose of allergen sensitization and challenge (8, 17, 28, 29).

In our work, we compare mice with BALB/c and C57BL/6 genetic background in their responsiveness to adjuvant-free epicutaneous sensitization and induction of food allergy (FA) to ovalbumin (OVA) by intragastric gavage. We compared the composition of the gut and skin microbiota of mice treated with OVA and that of PBS-treated controls after the epicutaneous sensitization and i.g. challenge. To elucidate the role of the skin and gut microbiota in the Th2 and mast cell immune response, we established this FA mouse model under the germ-free (GF) conditions.

## Materials and methods

### Animals

BALB/c and C57BL/6 mice (Institute of Microbiology, Nový Hrádek, Czech Republic) were housed in individually ventilated cages (Tecniplast S.P.A., Italy) in specific-pathogen-free (SPF) conditions, kept in a room with 12-h light-dark cycle at 22°C and fed *ad libitum* OVA-free mouse breeding diet V1124-300 (Ssniff Spezialdiäten GmbH, Germany), sterilized by irradiation ~25 kGy (Bioster, Czech Republic). The SPF condition was regularly checked by Federation of European Laboratory Animal Science Associations (FELASA). Germ-free (GF) female BALB/c mice were housed in plastic Trexler type isolators. Sterility was checked every three weeks by standard microbiological procedures to confirm the absence of aerobic and anaerobic bacteria, molds, and yeasts and by Gram staining of the fecal smears and inspection under the microscope. GF mice were maintained on a 12-h light–dark cycle with free access to autoclaved water and ~50 kGy (Bioster, Czech Republic) irradiated OVA-free diet V1124-300 (Ssniff Spezialdiäten GmbH, Germany), SAFE select fine bedding (Safe, Rosenberg, Germany) with enrichment nestlets (Plexx, Anlab, Czech Republic). This study was carried out in accordance with the recommendations of the Committee for the Protection and Use of Experimental Animals of the Institute of Microbiology Academy of Sciences of the Czech Republic (approval ID: 30/2020).

### Experimental design and induction of food allergy

**Figure 1:**
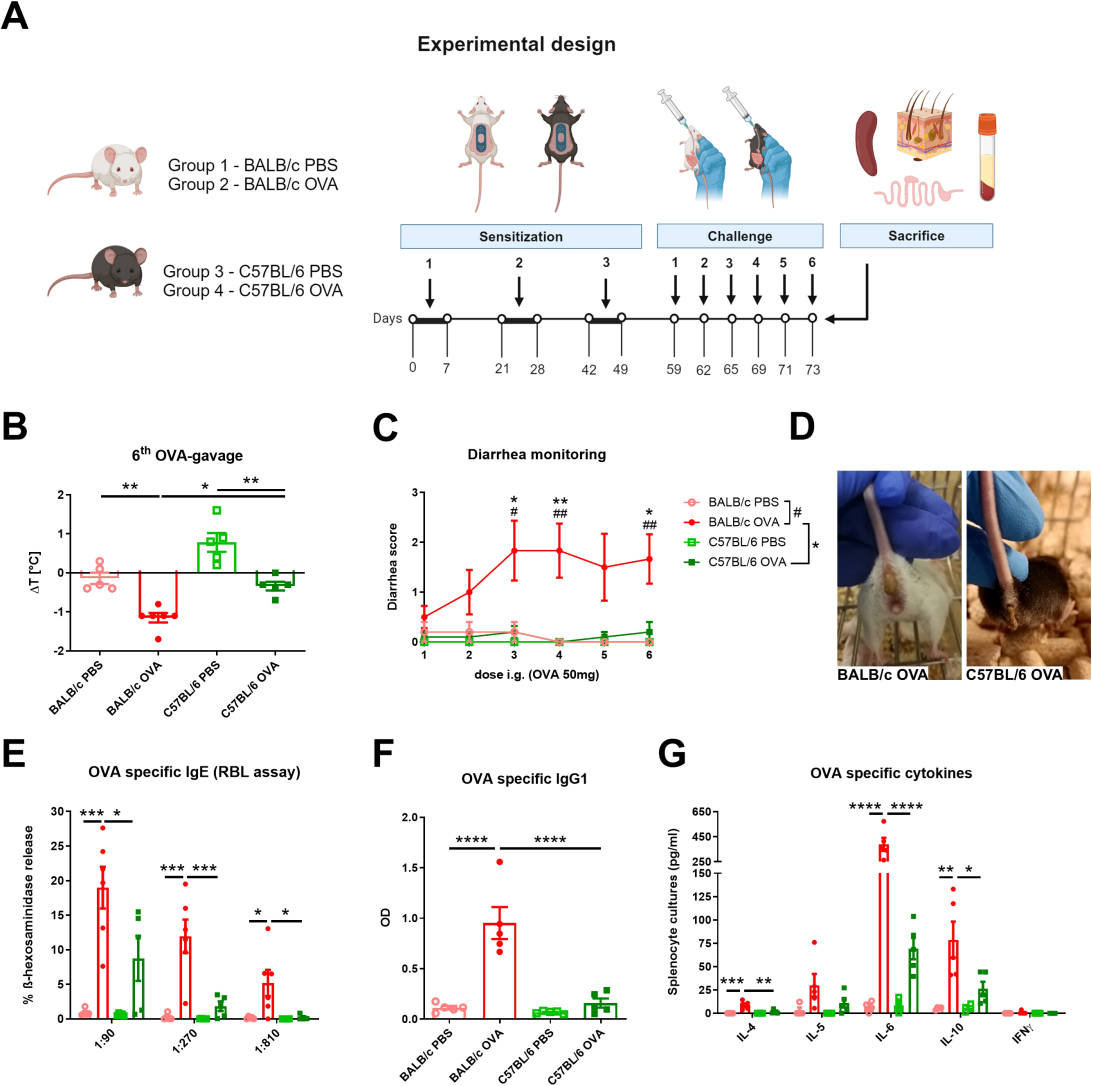
BALB/c and C57BL/6 mice differently respond to the adjuvant-free epicutaneous sensitization and intragastric challenge with ovalbumin. **(A)** A total of three one-week exposures of ovalbumin (OVA, 2 mg/ml) or PBS were applied as patches to shaved and tape-stripped skin of BALB/c and C57BL/6 mice, separated by two-weeks intervals. Ten days after the third epicutaneous sensitization, mice were intragastrically (i.g.) challenged by six doses of OVA (50 mg/200 µl PBS) or PBS (200 µl) alone. Experimental design was created by BioRender software. **(B)** The drop in rectal temperature was measured after the 6^th^ OVA-gavage in the mice. ΔT was determined as difference in the temperature before and 15 minutes after the challenge. **(C)** The occurrence of diarrhea was monitored 30 minutes after each OVA challenge. **(D)** Representative pictures of diarrhea occurrence in BALB/c OVA and C57BL/6 OVA-treated mice after the last i.g. OVA exposure. **(E)** OVA-specific IgE in serum was determined as β-hexosaminidase release from rat basophil leukemia cells (RBL). The results are expressed as % of total β-hexosaminidase release. **(F)** OVA-specific IgG1 in sera are expressed as optical density (OD). **(G)** Levels of IL-4, IL-5, IL-6, IL-10 and IFNγ were determined in OVA-stimulated splenocyte culture supernatants (pg/ml). Data are plotted as mean values ± SEM. One representative experiment out of two independent experiments is shown (BALB/c PBS n = 4-5, BALB/c OVA n = 5-6, C57BL/6 PBS n = 4-5, C57BL/6 OVA n = 5 mice per group). The significant difference between groups of mice was calculated by one-way ANOVA with Tukey´s multiple comparison test */# p <.0.05. **/^##^ p < 0.01. ***p < 0.001. ****p < 0.0001.

Eight weeks old female SPF mice were divided into four groups according to mouse strain and treatment: BALB/c PBS (n = 5); BALB/c OVA (n = 6); C57BL/6 PBS (n = 5); C57BL/6 OVA (n = 5). Epicutaneous (EC) sensitization of mice was done as previously described (30). Briefly, mice were anesthetized with Isoflurane (Aerrane, Baxter S.A., Belgium), dorsal skin was shaved with an electric razor and a razor blade and six-times tape-stripped by adhesive tape (3M, Scotch, Czech Republic). Altogether, 50 µl of ovalbumin in PBS (OVA; 2mg/ml) (grade V; Sigma Chemical Co., St. Louis, MO) or PBS alone was applied on a patch of sterile gauze, which was secured to the skin with a transparent bio-occlusive dressing (TegadermTM, 3M, St. Paul, USA) (**Supplementary Figure S1A**). The patch was re-applied to the same skin site third day and removed on day 7 of each EC exposure. Each mouse had a total of three one-week EC exposures to OVA or PBS separated by two-week rest intervals. Ten days after the last sensitization, mice were intragastrically gavaged (i.g.) three times a week for 2 weeks with 200 µl PBS with/without 50 mg OVA (experimental design, **Figure 1A**).

Eight weeks old germ-free female BALB/c mice (GF OVA, n = 7) and their SPF BALB/c counterparts (SPF OVA, n = 7) were anesthetized for 30 minutes by sterile solution of ketamine (2mg/200µl) and xylazine (0.2mg/200µl; both Bioveta, Czech Republic) in 0.9% NaCl, their hair was cut by scissors, the skin was shaved with sterile stainless razor and six-times tape-stripped with irradiated adhesive tape (3M, Scotch, Czech Republic). The EC sensitization and challenge of GF mice and their SPF counterparts was performed as described above with the sterile OVA filtered twice through a 0.22 µm filter (Corning, Germany) (experimental design, **Figure 4A**).

The occurrence of diarrhea in mice was assessed within 30 minutes after each OVA challenge as described by Schwarzer et al. (13) and the diarrhea score was evaluated according to the following criteria: 0 – normal, well-formed stool; 1 – soft, sticky well-formed stool; 2 – not formed stool; 3 – liquid diarrhea. After the last i.g. OVA or PBS gavage, drop in body temperature was measured by Thermocouple Thermometer with mouse rectal probe (World Precision Instruments Inc., USA) and determined as difference in the temperature before and 15 minutes after the challenge. Mice were sacrificed by cervical dislocation and sera, splenocytes, dorsal skin and small intestine samples were collected for further analysis. All experiments were repeated twice with variable number of mice per group.

### Humoral immune responses

Allergen-specific serum IgG1 levels were determined by ELISA as described previously (31). Briefly, Nunc™ MaxiSorp™ flat-bottom 96-well-plates were coated with ovalbumin (Worthington Biochemical Corporation; 5 µg/ml) and sera diluted 1/10000 were applied. Rat anti-mouse IgG1antibodies (1/500, Pharmingen, USA), peroxidase-conjugated mouse anti-rat IgG antibodies (1/2000; Jackson, Immuno Labs., USA) and ABTS solution (Sigma-Aldrich, USA) were used for the detection. Antibody levels were reported as optical density at wavelength λ = 405 nm. The activity of OVA-specific IgE in serum (diluted 1:90, 1:270 and 1:810) were measured by rat basophil leukemia (RBL-2H3) cells degranulation assay as described by Górska et al. (32). Briefly, RBL-2H3 cells were plated in 96-well tissue culture plates (4 × 10^4^ cells per well) and passively sensitized by incubation with mouse sera for 2 hours. Results were reported as a percentage of total β-hexosaminidase release from cells after disruption with 1% Triton X-100.

### Determination of cytokine production

Spleen cells (1 × 10^7^ cells/ml) were cultivated on 96-well flat-bottom plates in 200 µl of RPMI (Sigma-Aldrich, USA) supplemented with 10% Fetal Bovine Serum (Gibco, USA), 100 U/ml of penicillin, 100 µg/ml streptomycin, and 10 mM HEPES (Sigma-Aldrich, USA). Cells were left unstimulated or stimulated with OVA (100 µg/well; Worthington Biochemical Corporation, USA) and cultured for 72 h at 37°C/ 5% CO_2_. Supernatants were collected and stored at -40°C until further analysis. Levels of IL-4, IL-5, IL-6, IL-10, IL-13 and IFNγ were determined using a Mouse Cytokine/Chemokine Milliplex MAP Kit (Sigma-Aldrich, USA) and analyzed with a Bio-Plex 200 System (Bio-Rad Laboratories, USA).

### Histological evaluation of mast cells in jejunum and skin tissue

Skin and jejunal tissue specimens were fixed with 4% paraformaldehyde for 24-48 h and stored in 80% ethanol. Samples were processed as previously published (13). Briefly, deparaffinized 5 µm-thick sections were rehydrated in ethanol-to-water gradients and stained for chloroacetate esterase activity, which is characteristic of mast cells. After staining with reagent solution for 30 minutes in the dark, the sections were counterstained with hematoxylin for 2 minutes. Skin sections were stained by hematoxylin and eosin to determine the changes in the structure. The samples were viewed under Olympus BX 40 microscope equipped with an Olympus Camedia DP 70 digital camera, and the images were analyzed using Olympus DP-Soft (Soft Imaging System GmbH, Germany). The numbers of mast cells per 1 mm^2^ in jejunum or skin sections were determined.

### Jejunal homogenates

After aseptic removal of jejunum, homogenate was prepared. Lysis solution with 1% TRITON-X (Sigma-Aldrich, USA) and protease inhibitor (cOmplete Mini, Roche, Germany) was added to jejunum samples. After cooling on ice, the jejunum was homogenized for 2x30s/50 Hz using Tissue Lyzer and stainless-steel beads 7 mm (Qiagen, Gemany), frozen in liquid nitrogen, thawed, and homogenized again (3 cycles in total). Supernatants were collected after centrifugation and stored at -80°C. Protein content of the homogenates was determined by the Pierce™ BCA Protein Assay Kit (ThermoFisher Scientific, USA) using albumin as a standard. Levels of IL-4 and IL-13 in jejunal homogenates were measured by the Milliplex MAP Mouse Cytokine/Chemokine Panel (Sigma-Aldrich, USA) according to manufacturer’s instructions and analyzed with the Bio-Plex 200 System (Bio-Rad Laboratories, USA).

### ELISA for mast cell protease-1

Levels of mouse mast cell protease-1 (MCPT-1) in sera and jejunal homogenates were determined by commercial kit (Ready-SET-Go!^®^, eBioscience, USA) according to manufacturer’s instructions. MCPT-1 levels in jejunal homogenates are represented per 1 mg of total protein.

### RT-qPCR analysis of arachidonate 5-lipoxygenase and histidine decarboxylase genes expression

Total RNA was isolated from jejunum using the NucleoSpin RNA kit (Macherey-Nagel, Germany) according to the manufacturer´s instruction. Random primers and SuperScript II RT (Thermo Fisher Scientific, USA) were used to reverse-transcribe 1 µg of extracted RNA. qPCR reaction was performed using Luminaris HiGreen Fluorescein qPCR Master Mix (Thermo Fisher Scientific, USA) with Real-Time PCR Thermal Cycler qTOWER3G. Messenger RNA expression of target gene (*alox5, hdc*) was normalized to TATA box binding protein using the 2^−ΔCt^ method. Data were shown as fold induction to BALB/c PBS and GF OVA group, respectively. **Supplementary Table S1** contains a list of primers.

### Skin and stool sample collection and DNA extraction

Skin swabs and fecal samples were collected from BALB/c and C57BL/6 mice at the end of the experiment before the last i.g. administration of OVA and immediately frozen at -80°C. Total DNA from feces was isolated using the ZymoBIOMICS DNA Miniprep Kit (Zymo Research, Irvine, CA, USA) according to the manufacturer’s protocol. Dorsal skin samples were taken from a 4 cm^2^ area using a sterile flocked swab (FLOQSwabsTM COPAN Diagnostics INC., USA) soaked in sterile SCF-1 buffer, as previously described (33). Extraction of total DNA from swabs was performed using the DNeasy PowerBiofilm kit (Qiagen, Germany) with minor modifications to the protocol as previously described (34).

### PCR amplification, sequencing, and data analysis

The subsequent PCR amplification of bacterial DNA was performed with degenerate primers 341F and 806R, which target the V3V4 region of 16S rRNA, as previously described (34). Briefly, a 25 μl reaction mixture was prepared for each sample in triplicates. PCR amplification was performed using 1X HiFi polymerase (Roche, USA), 0.4 μM primers, and 5 μl of template. Thermal cycling parameters started with an initial hold step of 95°C for 3 min, followed by 33 cycles of denaturation (94°C, 3 min), annealing (55°C, 5 s), and extension (72°C, 2 min) for skin samples or by 25 cycles of denaturation (95°C, 30 s), annealing (55°C, 30 s), and extension (72°C, 30 s) for fecal samples and finalized by an elongation step at 72°C for 5 min. Triplicates of PCR products were pooled to minimize random PCR bias, and the correct length of amplicons was verified by agarose gel electrophoresis. PCR amplification negative controls, extraction and sequencing positive controls (mock communities; ZymoBIOMICS Microbial Community Standard and ZymoBIOMICS Microbial Community DNA Standard, Zymo Research, USA; both in linear and logarithmic form) were processed in a similar manner.

The PCR amplicons were processed as previously described (34). Briefly, the pooled amplicons were normalized using a Sequal-Prep™ Normalization Plate Kit (Illumina, USA). Adapters compatible with the MiSeq platform were ligated using the KAPA HyperPrep kit (Roche, USA), quantified, and sequenced using the MiSeq Reagent Kit v2 (2 x 300 bp) at the CEITEC Genomics Core Facility (Brno, Czech Republic).

Quality control of raw data was observed using FastQC (version 0.12.0). Trimming was performed using Cutadapt (version 2.10) and trimmed reads were merged using Fastq-join (version 1.3.1) with default parameters. After demultiplexing with custom R script, samples were imported into QIIME2 (35) (version 2022.2), where the table with ASVs was generated by DADA2 plugin. Rarefaction depth was set to a sample size of the minimal sequencing depth where majority of species have been observed within a given number of samples. For bacterial taxonomy assignment, BLAST classifier and SILVA 138 database with 99% similarity were used. Mock community samples were employed as a positive control and processed along with samples. Different treatments and mouse strains were compared within QIIME2. Contaminating taxa (chloroplasts, mitochondria) as well as taxa with less than 1% abundance in less than 20% of samples were removed from skin samples analysis. β-diversity data matrix obtained from QIIME2 was visualized in R using QIIME2R package (version 0.99.6) (36). The sequencing data are available at the Sequencing read archive under accession number PRJNA1145657.

### Statistical analysis and data visualization

Data are expressed as mean ± standard error of mean (SEM). One-way ANOVA with Tukey’s multiple comparison test or unpaired t-test were used for comparison between experimental groups of mice. Statistical analysis was performed using GraphPad Prism Software (version 9.4.1; San Diego, USA). Experimental design was created by BioRender online software.

The α-diversity results for Shannon entropy, Chao1 index, Faith´s phylogenetic diversity and Observed ASVs, which we obtained from QIIME2, were visualized in GraphPad Prism (version 9.4.1) and compared with One-way ANOVA non-parametric test. The β-diversity was analyzed within QIIME2. PC1 and PC2 axes represent the major variability. Clustering is illustrated using the mean (centroid) of each group of samples. Centroids relate to individual samples using lines. Comparison in β-diversity for BALB/c versus C57BL/6 and PBS versus OVA treated groups was done using PERMANOVA test with 999 permutations, where *p-*values and *q-*values calculated with Benjamini and Hochberg correction are shown (37). Rarefied data were used for bar plot visualization (number of reads for fecal samples 1489 and skin swabs 1700) and rarefied curves are shown in **Supplementary Table S2**.

Heatmap was created in R (ver. 4.4.1) (38) using pheatmap package (ver 1.0.12) (39), the values were standardized and are presented as z-score. Reduction of dimensionality of multivariate data was performed using the principal component analysis (PCA) in R using FactoMineR (ver. 2.11) (40) and factoextra packages (ver. 1.0.17) (41). PCA is presented as biplot showing 1^st^ and 2^nd^ principal component.

## Results

### Mouse genetic background drives the sensitivity to epicutaneous sensitization and food allergy

BALB/c and C57BL/6 mice were challenged six-times by intragastric gavage of OVA over the course of two weeks after adjuvant-free epicutaneous sensitization to OVA (**Figure 1A**). OVA challenges led to the development of experimental FA symptoms only in EC sensitized BALB/c mice, characterized by a drop in core body temperature after the last OVA gavage (**Figure 1B**) and occurrence of diarrhea as early as the second OVA gavage (**Figures 1C, D**). Further, OVA-immunization led to significant induction of OVA-specific antibodies in serum of BALB/c OVA-treated mice compared to C57BL/6 OVA-treated mice and PBS controls. Increased levels of OVA-specific IgE (determined by RBL assay) correlated with increased levels of OVA-specific IgG1 (**Figures 1E, F**). In addition, recall OVA-induced cytokine secretion (IL-4, IL-6, and IL-10) by spleen cells was markedly altered in BALB/c OVA-treated mice compared to C57BL/6 OVA-treated mice and BALB/c PBS-treated control group. On the other hand, there was a non-significant increase in OVA-induced IL-5, IL-6 and IL-10 levels between OVA-treated C57BL/6 mice and the C57BL/6 PBS control group. We did not observe elevated levels of the Th1-related cytokine IFNγ in any experimental group (**Figure 1G**).

### High numbers of jejunal mast cells revealed in BALB/c but not C57BL/6 OVA-treated mice

**Figure 2:**
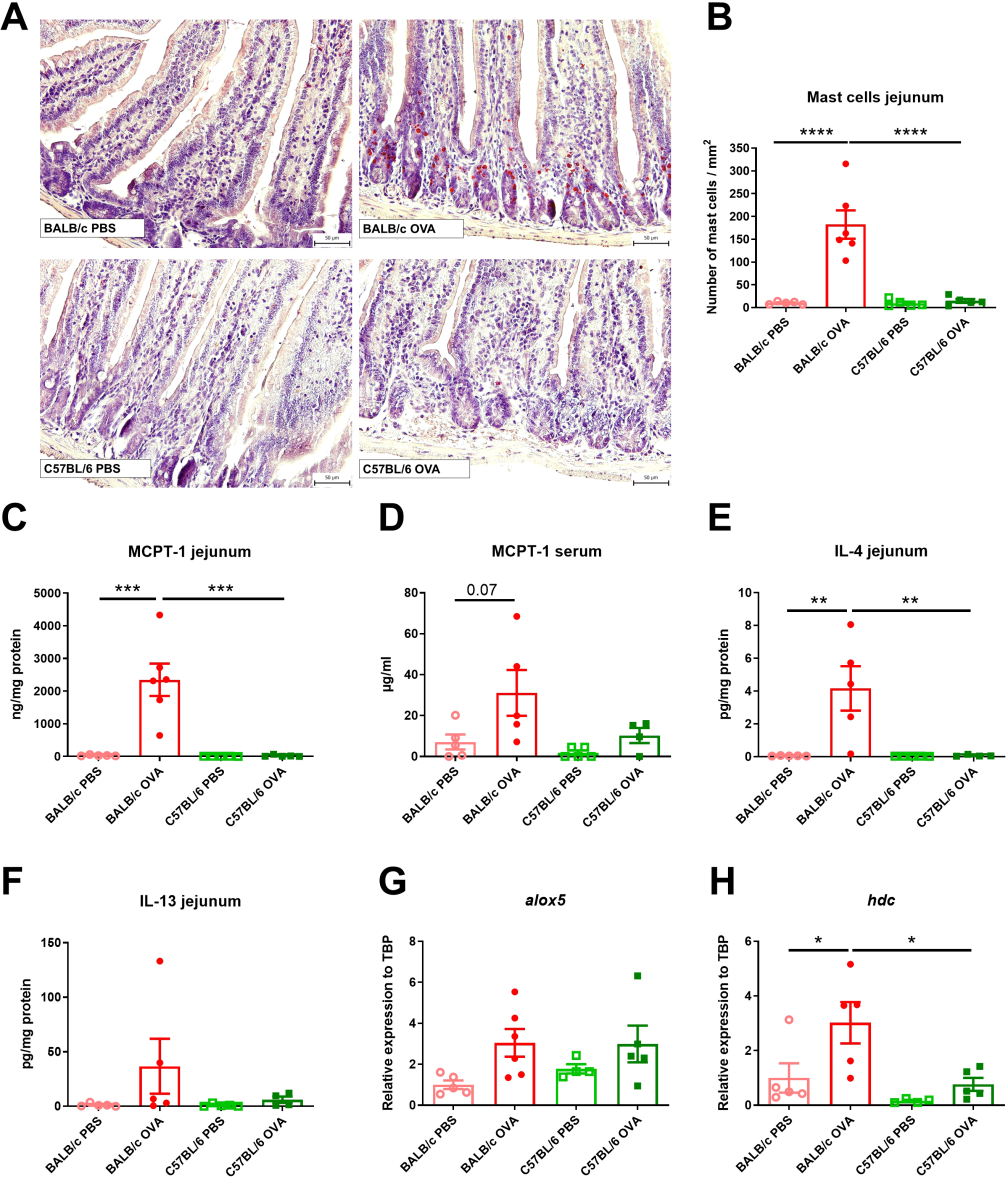
BALB/c, but not C57BL/6 mice, display high numbers of mast cells and Th2 response in jejunum after adjuvant-free epicutaneous sensitization and intragastric challenge with ovalbumin. **(A)** Histological staining of mast cells in jejunal sections by hematoxylin/pararosaniline was performed on samples from PBS controls and OVA-treated mice (scale bars 50 μm). **(B)** Quantification of mast cells per 1 mm^2^ in jejunal sections. MCPT-1 levels were determined in **(C)** jejunal homogenates (ng/mg protein) and **(D)** serum (µg/ml) by ELISA. Levels of **(E)** IL-4 and **(F)** IL-13 in homogenate of jejunum were expressed as pg/mg of protein. Expression levels of **(G)** *alox5* and **(H)** *hdc* in the jejunal tissues is shown as fold change relative to the BALB/c PBS group. Data are plotted as mean values ± SEM. Values of one representative experiment out of two independent experiments are shown (BALB/c PBS n = 5, BALB/c OVA n = 5-6, C57BL/6 PBS n = 4-5, C57BL/6 OVA n = 4-5 mice per group). The significant difference between groups was calculated by one-way ANOVA with Tukey´s multiple comparison test *p < 0.05; **p < 0.01; ***p < 0.001; ****p < 0.0001.

Elevated numbers of mast cells (MC) in small intestine are a hallmark of food allergy, both in humans and mice (13). OVA-treated C57BL/6 mice had similar counts of MCs in jejunum compared to control mice (PBS-treated BALB/c and C57BL/6 mice, **Figures 2A, B**). On the other hand, BALB/c OVA-treated mice showed profound infiltration of mucosal MCs to the jejunal tissues, accompanied by elevated levels of MCPT-1 in the intestinal tissue homogenates and sera (**Figures 2C, D**). Concomitantly, the local production of Th2-associated IL-4 and IL-13 was significantly higher in the jejunum of OVA-treated BALB/c mice compared to OVA-treated C57BL/6 mice and PBS controls (**Figures 2E, F**). We examined the jejunal expression of two genes, arachidonate 5-lipoxygenase (*alox5*) and histidine decarboxylase (*hdc*), which are known to regulate leukotriene and histamine production in mast cells (42). We observed a trend towards upregulation of *alox5* in BALB/c and C57BL/6 OVA-treated mice (**Figure 2G**) and significant upregulation of *hdc* (**Figure 2H**) genes in jejunum of BALB/c OVA-treated mice only.

Skin of BALB/c OVA mice showed mild signs of atopic dermatitis with continuous layer of thin epidermis and slightly increased number of cells in skin structures compared to C57BL/6 OVA mice and PBS controls (**Supplementary Figure S1B**). On the other hand, we did not observe any increase in the number of mast cells in the skin among the experimental groups (**Supplementary Figures S1C, D**).

### The composition and diversity of the gut microbiome depends on both genetic background and OVA-treatment

**Figure 3:**
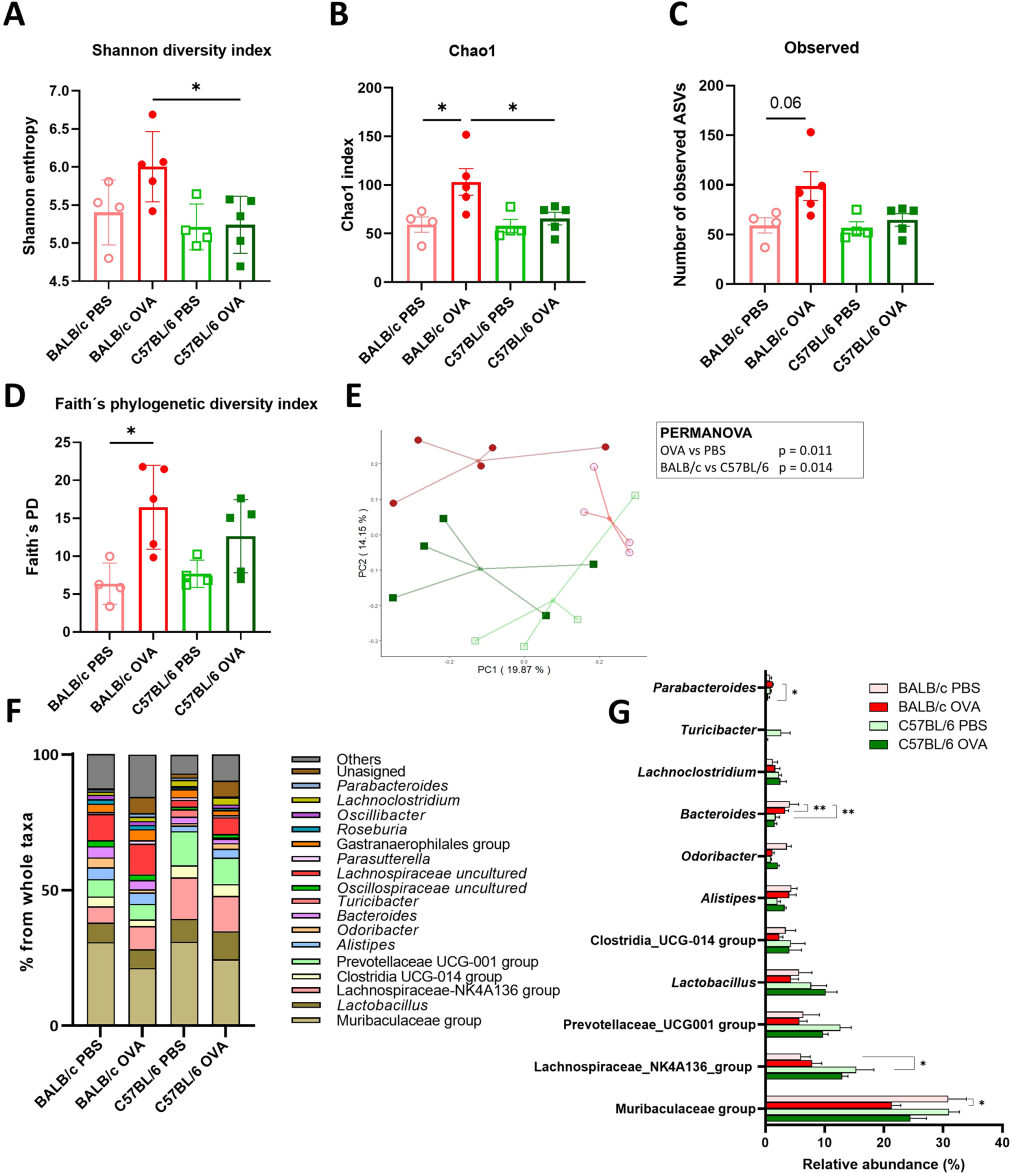
Microbiome analysis revealed the differences in the composition and diversity of fecal bacteria in BALB/c mice after the EC sensitization and food allergy induction. The α-diversity is represented by **(A)** Shannon diversity index, **(B)** Chao1 index, **(C)** Observed ASVs and **(D)** Faith’s phylogenetic diversity index. **(E)** PCA plot showing β-diversity represented by the unweighted UniFrac distances among samples in studied cohorts. Each point shows individual sample (BALB/c PBS - red empty circles, BALB/c OVA - red filled circle, C57BL/6 PBS - green empty square, C57BL/6 OVA - green filled square). The association between study groups was tested using the PERMANOVA test. **(F)** Taxonomy bar plot of microbiome composition in stool samples is shown at the genus level. Bars represent average abundance in percentage of taxa for all samples within a group. Taxa with less than 1% abundance are shown as “Others”. **(G)** Statistical comparison of the highly abundant bacterial taxa is shown for all experimental groups. Significance between groups was determined by One-way ANOVA with Tukey´s multiple comparison test, *p < 0.05; **p < 0.01.

It has been implicated that changes in microbiota go hand in hand with FA severity in allergic patients (4). To better understand how epicutaneous sensitization and intragastric challenge by OVA affected the composition of gut (**Figure 3**) and skin (**Supplementary Figure S2**) microbiota, we performed 16S rRNA sequencing analysis of the feces and skin swab from the experimental mice. Bacterial α-diversity characterized by Shannon diversity index, Chao1 index and numbers of observed ASVs was significantly increased in feces of BALB/c OVA mice compared to C57BL/6 OVA or BALB/c PBS mice (**Figures 3A-C**). Similarly, Faith’s phylogenetic diversity index was significantly increased in BALB/c OVA mice compared to PBS group (**Figure 3D**) showing higher abundance of phylogenetically related species in the fecal samples after OVA treatment in BALB/c mice only. Further, unweighted UniFrac PCA with PERMANOVA analysis revealed that gut microbiome of experimental mice exhibited distinct clustering based on both genetic background (p = 0.014) and the OVA treatment (p = 0.011) (**Figure 3E**). Relative abundance of bacterial genera is shown as percentage from whole taxa depicted by taxonomy bar plot (**Figure 3F**; **Supplementary Figure S2F**). The microbiome analysis of fecal samples revealed that majority of detected bacteria belongs to the Muribaculaceae, Lachnospiraceae and Prevotellaceae groups and genus *Lactobacillus* whereas members of genus *Bacteroides* or *Parabacteroides* remained in minority (**Figures 3F, G**). Interestingly, the Lachnospiraceae-NK4A136 or Prevotellaceae groups were more prevalent in fecal samples of C57BL/6 mice compared to BALB/c mice. In the BALB/c mice, epicutaneous sensitization and intragastric challenge with OVA significantly decreased the relative abundance of Muribaculaceae group and genus *Odoribacter* in comparison to PBS group (**Figure 3G**).

**Figure 4:**
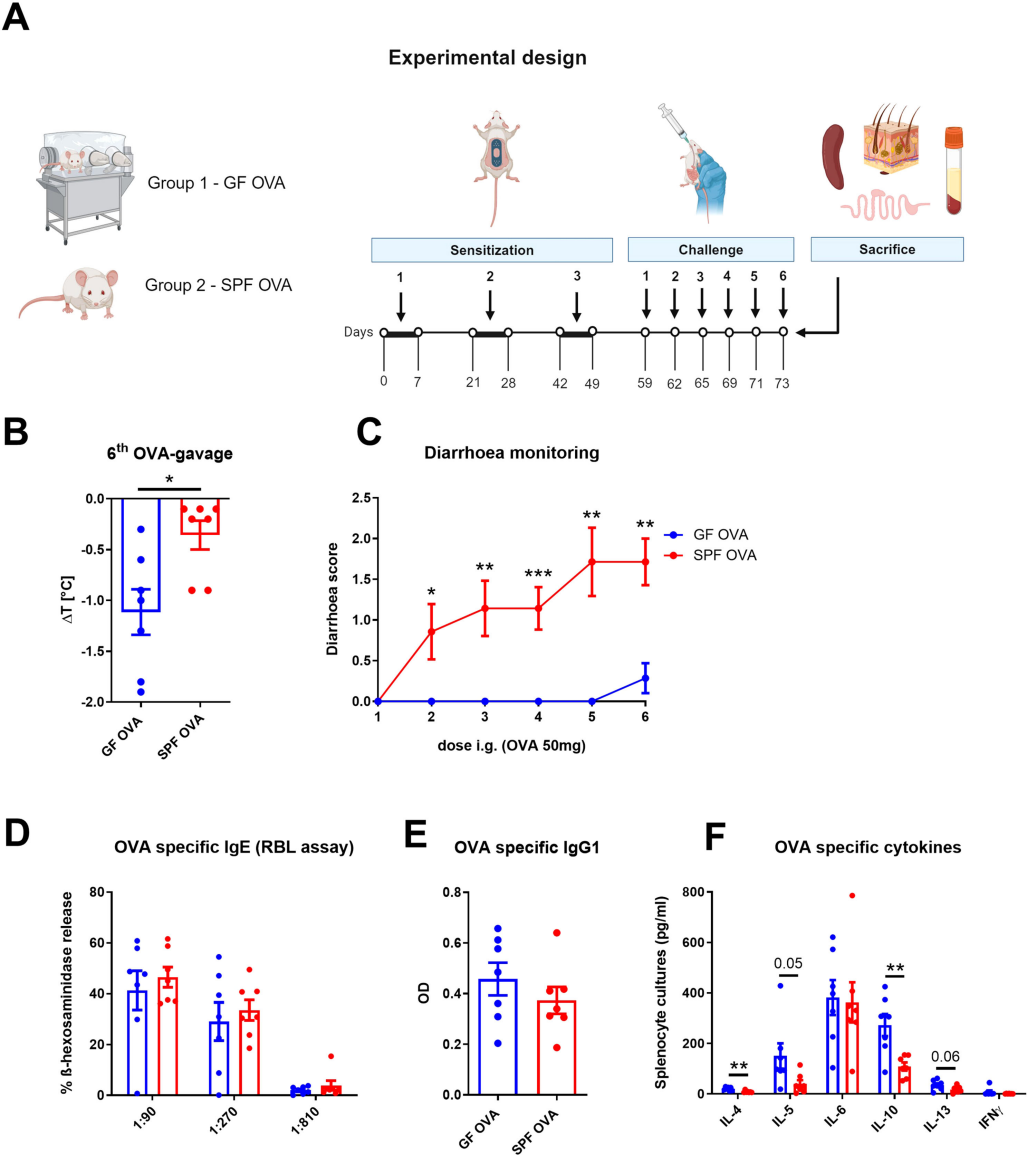
Adjuvant-free epicutaneous sensitization and intragastric challenge with ovalbumin led to decreased temperature but not diarrhea in GF mice. **(A)** Experimental design: A total of three one-week exposures to ovalbumin (OVA, 2 mg/ml) were applied as patches to shaved and tape-stripped skin of GF and SPF mice, separated by two-week interval. Ten days after the third epicutaneous sensitization, mice were intragastrically (i.g.) challenged by six doses of OVA (50 mg/200 µl PBS). Experimental design was created by BioRender software. **(B)** The drop in rectal temperature was measured after the 6^th^ OVA gavage in the mice. ΔT was determined as difference in the rectal body temperature before and 15 minutes after the last OVA challenge. **(C)** The occurrence of diarrhea was monitored 30 minutes after each OVA challenge. **(D)** OVA specific IgE was measured by β-hexosaminidase release from rat basophil leukemia cells (RBL) in mouse sera diluted to 1:90, 1:270 and 1:810. The results were expressed as % of total β-hexosaminidase release. **(E)** OVA-specific IgG1 in sera was measured by ELISA and expressed as optical density (OD). **(F)** Levels of IL-4, IL-5, IL-6, IL-10, IL-13 and IFNγ were determined in OVA-stimulated splenocyte culture supernatants. Values are shown after subtraction of cytokine levels determined in unstimulated splenocyte cultures (pg/ml). Data are plotted as mean values ± SEM. Values of one representative from two independent experiments (GF OVA, n = 7; SPF OVA, n = 7 mice per group) are shown. Unpaired t-test was used for comparison between experimental groups of mice. *p < 0.05; **p < 0.01; ***p < 0.001.

In the skin microbiome analysis, we detected no specific differences in α-diversity (Shannon diversity index, Chao1 index, Observed ASVs) among the experimental groups (**Supplementary Figures S2A-C**). PCA plot showing β-diversity is represented by the unweighted and weighted UniFrac distances among samples in cohorts (**Supplementary Figures S2D, E**). Using PCA and PERMANOVA analysis we determined clear clustering of skin microbiome β-diversity based on genetic background of the mice (BALB/c *vs* C57BL/6, p = 0.001), whereas the OVA-treatment did not change the diversity parameters. Using taxa bar plot, we showed high variety of the bacterial genera with their relatively low abundance in the skin swab samples (**Supplementary Figure S2F**). Microbiome analysis revealed similarly high relative abundance of the anaerobic bacteria of Muribaculaceae, Lachnospiraceae or Clostridia groups naturally inhabiting the mouse intestine suggesting the common contamination of the mice skin from the bedding environment. Moreover, the relative abundance of these contaminating bacteria seems to be dependent on mouse genetic background with the elevated abundance of Muribaculaceae in BALB/c mice and Lachnospiraceae in C57BL/6 mice. Interestingly, we found elevated abundance of genus *Staphylococcus* in C57BL/6 mice (regardless the treatment), but no presence of this genus in BALB/c OVA-treated mice. On the other hand, the relative abundance of skin commensals, such as *Acinetobacter*, *Streptococcus*, *Enhydrobacter* or *Aerococcus*, revealed no significant differences neither between BALB/c versus C57BL/6 mice nor between OVA-treated versus PBS-treated mice (**Supplementary Figure S2G**).

### Multivariate analysis of microbiota and immune response showed a clear separation of BALB/c OVA group

Using multivariate analysis, we investigated the relationship between gut or skin microbiota abundance and the main parameters of immune response to food allergen, as anaphylactic hypothermia and diarrhea, cytokine response in splenocytes (IL-4, IL-5, IL-10 and IL-13), number of mast cells and cytokine response in jejunum (IL-4, IL-13, MCPT-1). Using the heatmap, we clearly distinguished the group of BALB/c OVA mice from clusters of BALB/c PBS and both C57BL/6 PBS and C57BL/6 OVA mice (**Supplementary Figure S3A**). Interestingly, the immunological response of the jejunum as IL-4, IL-13, MCPT-1 and MCs created distinct cluster with the bacterial genus *Alistipes*. However, multivariate analysis did not reveal distinction between skin and gut bacteria clusters showing the narrow relation of the skin and gut microbiome in the experimental mice. Using the PCA analysis (**Supplementary Figure S3B**), we depicted the contribution of the immunological factors or bacterial taxa to the separation of mouse experimental groups. We confirmed that the immunological responses such as diarrhea, cytokine response in splenocytes and jejunum, the number of mast cells as well as the bacteria on the skin (i.e. Muribaculaceae group, *Erysipetoclostridium*, *Staphylococcus*, Rikenellaceae RC9 group) mainly contributed to the conclusive separation of BALB/c OVA group in this analysis.

### Germ-free mice show enhanced allergy response to OVA but do not develop diarrhea

Having established that OVA treatment alters the gut and skin microbiome composition in EC sensitized BALB/c mice, we aimed to determine the role of gut and skin microbiome in the induction of allergic response by EC OVA-sensitization and i.g. challenge in BALB/c mice. To test the role of the microbiota, we compared SPF and GF BALB/c mice in the same experimental model (**Figure 4A**). We found that OVA-sensitized and challenged SPF mice developed symptoms of FA characterized by a drop in body core temperature and diarrhea occurrence (**Figures 4B, C**). Sensitized GF mice showed a 3.12-times greater decrease in temperature compared to SPF mice (**Figure 4B**). SPF mice developed allergic diarrhea as early as after the second OVA gavage, while GF mice were unable to develop allergic diarrhea at all (**Figure 4C**). Interestingly, we found no differences in the levels of OVA-specific IgE and IgG1 in serum of SPF and GF OVA-treated mice (**Figures 4D, E**). We further evaluated the effect of microbiota on recall OVA-induced cytokine response from OVA-sensitized mouse splenocytes. The levels of IL-4 and IL-10 were significantly increased in GF OVA-treated mice when compared to SPF OVA-treated mice. The same trend was also observed for IL-5 and IL-13 cytokine levels (**Figure 4F**). Thus, GF mice are prone to stronger systemic allergic response presented by anaphylactic hypothermia and allergen specific Th2-cytokine induction in spleen after EC OVA-sensitization and challenge, while some symptoms of allergy, such as diarrhea, seems to be microbiota-dependent.

### Germ-free mice have higher mast cells response in the jejunum, but not in the skin after EC sensitization and OVA challenge

**Figure 5:**
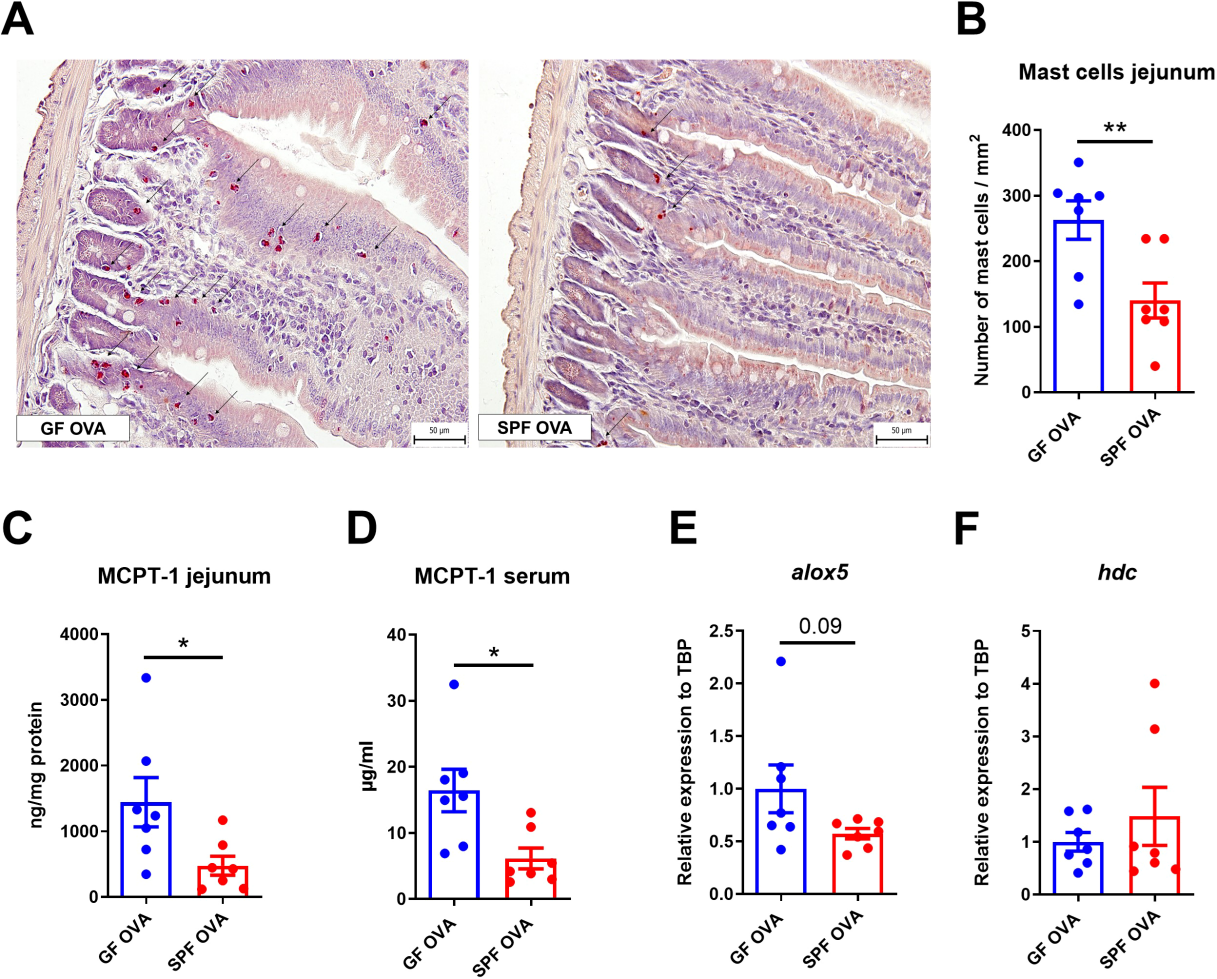
Germ-free BALB/c mice show high numbers of mast cells in jejunum after adjuvant-free epicutaneous sensitization and intragastric challenge with ovalbumin. **(A)** Representative picture of mast cells staining by hematoxylin/pararosaniline in jejunal sections from OVA-treated GF and SPF BALB/c mice (scale bars, 50 μm). **(B)** Quantification of mast cells per 1 mm^2^ of tissue in jejunal sections. Mast cell protease-1 (MCPT-1) levels were determined in **(C)** jejunal homogenates (ng/mg protein) and **(D)** sera (µg/ml) by ELISA. Expression levels of **(E)** *alox5* and **(F)** *hdc* in the jejunal tissues are shown as fold change relative to the BALB/c PBS group. Data are plotted as mean values ± SEM. Values of one representative experiment out of two independent experiments (GF OVA n = 7, SPF OVA n = 7 mice per group) are shown. Unpaired t-test was used for comparison between experimental groups of mice. *p < 0.05; **p < 0.01.

OVA-treated GF mice have significantly more mast cells in the jejunum than SPF mice. Moreover, MC in GF mice were distributed in both the crypt and the lower part of the intestinal villi, whereas MC in SPF mice were found only in the crypt base (**Figures 5A, B**). Similarly, MCPT-1 levels in jejunal homogenates and sera were significantly higher in GF mice compared to SPF mice (**Figures 5C, D**). Consistently, we detected increased *alox5* gene expression in the jejunum of GF mice (**Figure 5E**), but no difference in *hdc* mRNA jejunum samples between the experimental groups (**Figure 5F**). We found no differences in the number of MCs in the skin between GF and SPF mice and only a small cell infiltration into the dermis compartment showing negligible changes in skin histology (**Supplementary Figures S4A, B**).

## Discussion

Skin sensitization can trigger an immune response that predisposes individuals to allergic reactions, indicating a crucial role of the skin barrier in the initiation and progression of allergic diseases (43–45). However, different mouse model of allergen skin sensitization lead to a different immune responses, depending on the mouse strain, route of sensitization, type and dose of allergen, or mechanism of skin barrier disruption (11). To date, many mouse models have been generated for food allergy research utilizing both wild-type and genetically-modified BALB/c and C57BL/6 mouse strains (8, 46–48). The immunodeficient mice are also utilized in so called humanized mouse models generated by engrafting human cells or tissues, that overcome some of the disparities between murine and human immunology and offer the unique possibility to investigate highly complex human allergic responses. This approach enable us to study the multistep allergic mechanisms as well as the impact of novel treatment options (16, 28, 49). Although BALB/c mice are a preferable model for allergic disease due to their Th2-biased responses (18, 50–52), some authors have shown effective responses in both BALB/c and C57BL/6 strains (8, 22, 23, 53–56). And while BALB/c and C57BL/6 mice are equally prone to allergic asthma model when immunized intraperitoneally, only the latter has been shown to respond with airway hyperresponsiveness to methacholine when sensitized epicutaneously (57). In our study, we compared the effects of epicutaneous, adjuvant-free, sensitization and intragastric challenge with ovalbumin (OVA) on the manifestation of FA symptoms in BALB/c and C57BL/6 mouse strains.

We found that BALB/c mice, but not C57BL/6 mice, showed clinical signs of FA (hypothermia, diarrhea) as well as allergen-specific IgE and IgG1 in serum and Th2 polarizing cytokines in spleen and jejunum after epicutaneous sensitization and OVA challenge. Moreover, OVA-treated BALB/c mice have increased mast cell numbers, higher MCPT-1 production and histidine decarboxylase (*hdc*) mRNA expression in the jejunum, as compared to either OVA-treated C57BL/6 or control mice. Food antigens administration through the defective skin barrier usually results in robust antigen-specific IgE production, intestinal mast cell expansion and anaphylaxis with diarrhea upon oral allergen challenge in BALB/c mice (58–60). Contrary, recent study describes that C57BL/6 mice, rather than BALB/c mice, may be better suited for specific epicutaneous sensitization and a food allergy model than BALB/c mice due to the stronger Th2-biased response and more severe disruption of the intestinal barrier (8). In contrast to our study, these studies mentioned above utilized mice cutaneously pretreated with MC903 (calcipotriol), a vitamin D3 analogue inducing skin hyperkeratosis, pruritis and type 2 inflammation that mimics AD lesions. MC903 is able to increase TSLP in the skin and induce mild MC infiltration to the jejunum of BALB/c mice on its own (60), which may mask the natural tendency of BALB/c mice to respond with a Th2 biased response. On the other hand, disruption of the skin barrier by tape stripping alone may cause IL-33 production from keratinocytes in skin of both BALB/c and C57BL/6 mice that further stimulates the ILC2 cells in the intestine to the expression of IL-4 and IL-13 and subsequent intestinal mast cell expansion (20). However, unlike in OVA-treated BALB/c mice, jejunal IL-4 and IL-13 in shaved, tape-stripped and PBS-treated mice were still mostly below detection limit in our experiments, suggesting that tape stripping alone has only limited effect. The shift in Th2 cytokines accompanied by significantly higher production of MCPT-1 as well as more than 8-fold higher number of intestinal MCs was found in BALB/c OVA-treated mice only compared to C57BL/6 OVA-treated mice and PBS control groups. Moreover, increased mRNA expression of 5-lipoxygenase and histidine decarboxylase in jejunum of BALB/c OVA-treated mice indicates the activation and effector function of their jejunal MCs. These data are in line with Nagashima et al. (24) who demonstrated that mast cells from BALB/c mice showed higher degranulation capacity compared to those from C57BL/6 mice and their results suggest the importance of carefully considering the choice of mouse strain when conducting experiments involving mast cells.

Gut or skin microbiota dysbiosis can cause significant epithelial barrier dysfunction which facilitates the allergen sensitization (61, 62). We (13) and others (12) showed that the skin microbiome undoubtedly influences the MCs recruitment, maturation and function in the dermis. In this study, we detected no specific differences in α-diversity of the skin microbiome among the experimental groups. Analysis of skin microbiome β-diversity revealed clear clustering based on genetic background of the mice (BALB/c or C57BL/6) regardless the OVA-treatment. Furthermore, we showed high variety of bacterial genera with their relatively low abundance in skin swabs. We found that the most abundant taxa on the mice skin are anaerobic bacteria naturally colonizing the intestine of mice, such as Muribaculaceae, Lachnospiraceae, Prevotellaceae or Clostridia groups, suggesting the commonly occurring contamination of the mice skin from the bedding environment. The relative abundance of these contaminating bacteria seems to be dependent on mouse genetic background with the elevated abundance of Muribaculaceae in BALB/c mice and Lachnospiraceae in C57BL/6 mice. Whether or how these “contaminating” bacteria could specifically contribute to immunological response in the skin of EC sensitized mice would needs to be further determined. It has been shown previously that *Staphylococcus aureus* is the bacterium with profound implication of eczema lesions severity in atopic dermatitis patients and up-regulated allergic sensitization through the disrupted skin (63). Interestingly, we found elevated abundance of genus *Staphylococcus* in C57BL/6 mice (regardless the treatment) but no presence in BALB/c OVA-treated mice, which manifested symptoms of food allergy, suggesting that presence of these specific bacteria do not play the significant role in EC sensitization in this experimental model. Rodriguez et al. (64) described correlation between colonization by staphylococci at the site of allergen sensitization and reduction in severity of the allergic response. On the other hand, the relative abundance of skin commensals, such as *Acinetobacter*, *Streptococcus*, *Enhydrobacter* or *Aerococcus* did not significantly differ neither between BALB/c versus C57BL/6 mice nor between OVA-treated versus PBS-treated mice. The analysis of the skin microbiome is in line with occurrence of mast cells in the skin of both BALB/c and C57BL/6 mice showing that 4 weeks after the last epicutaneous OVA application, immunological parameters as well as skin microbiome were unchanged and depend more on genetic background than on allergen treatment.

Contrary to skin, changes in gut microbiota of BALB/c mice were specifically dependent on both genetic background and OVA treatment. The α-diversity of the intestinal microbiome in BALB/c OVA-treated mice differed from PBS controls and C57BL/6 OVA mice suggesting that development of FA symptoms after five i.g. doses of OVA (diarrhea and anaphylactic hypothermia) could influence the specific microbiome composition in the feces. Individuals with food allergies commonly exhibit reduced richness and diversity of the gut microbiota described in both allergic patients (59) and experimental mice (8, 65–69). In contrast, we have shown increased α-diversity of intestinal microbiome expressed by Shannon entropy and Faith´s index that could be caused by reduction of highly abundant taxa and appearance of the new taxa which are normally under the detection level. Interestingly, the β-diversity revealed that composition of microbiome is specifically dependent on both, genetic background and OVA treatment. Further, we found significant differences in the relative abundance of Muribaculaceae, Lachnospiraceae-NK4A136 and Prevotellaceae groups of bacteria and genus *Odoribacter* among the experimental groups. Lachnospiraceae NK4A136 are members of the clostridial cluster XIVa of the phylum Firmicutes. The Lachnospiraceae family comprises strictly anaerobic bacteria, which belong to the core microbiota as one of the most abundant in the human and murine gut. Reduction in Lachnospiraceae within the gut microbiota has been associated with variety of pathological of conditions, including allergies, inflammatory bowel disease, and metabolic disorders (68, 70). Gu et al. (68) showed that the gut microbiota composition was reshaped in peanut-allergic mice, with Lachnospiraceae_NK4A136_group significantly down-regulated and Muribaculaceae group up-regulated. On the contrary, decrease in abundance of *Muribaculaceae* spp. has been described with the OVA sensitization in BALB/c (69) or C57BL/6 (8). Recent work showed that antibiotic-induced dysbiosis in the microbiome promoted the development of food allergy and was associated with decreased abundance of Muribaculaceae representatives. Members of family Muribaculaceae are usually found as dominant in healthy mouse microbiota within the gut where they degrade polysaccharides to generate SCFAs such as succinate, acetate and propionate (71). Prevotellaceae members, SCFAs producers from dietary fiber, are associated with the intestinal microbiota of healthy individuals, mainly in rural environment and their numbers are decreased in allergic patients (71). Bacteria of the genus *Odoribacter* has been shown to contribute to the increase of SCFAs in the intestine (acetic acid, propionic acid, and succinic acid) and to protect against intestinal inflammation and colon cancer with immunoregulatory effects (72). Along these lines, accumulating evidence supports the role of microbiota-derived SCFAs in promoting tolerogenic immune responses in the healthy intestine and specifically affecting the MCs by direct inhibition of the IgE-mediated mast cell degranulation and allergen-induced histamine release, thus preventing the symptoms of allergic reaction (73).

Similarly, Feehly et al. (74) described the correlation of commensal bacteria from healthy donors and the regulation of food allergy responses in mouse recipients. They showed a clear separation of the composition of fecal bacteria in healthy and allergic mice and found that the majority of the bacterial taxa changed in the same direction (increase or decrease in abundance) in both fecal and ileal samples. Based on the correlation of ileal bacteria with the upregulated genes of healthy or allergic mice, they identified bacterium *Anaerostipes caccae*, which belongs to genus *Clostridium*, to be involved in the protective response against food allergy. In our study, we described the differences in the fecal microbiota composition of mice that suffered from short periods of diarrhea after each ovalbumin challenge (BALB/c OVA group), which could dynamically influence the homeostasis of the microbiota population in colon. Using multivariate analysis, we summarized the differences in the gut and skin microbiome with key immunological characteristics and described their contribution to the cluster analysis of the experimental groups. Similar to Feehley et al. (74), we clearly distinguished the mice with allergic reaction (BALB/c OVA) from groups of healthy mice. Using a heatmap, we were able to illustrate the association between the genus *Alistipes* and immunological parameters such as mast cells or cytokine response in jejunum. Previously, in a different mouse model, the genus *Alistipes* has been shown to positively correlate with IL-6 production and mast cell proliferation, and associated with the colorectal cancer progression in experimental mice (75). Nevertheless, further research is needed to show the contribution of these bacteria to the mast cell response and the development of allergy or to the general changes in the composition of bacteria in jejunum as the main site of allergic mast cell response.

Given that OVA treatment altered the composition of the gut microbiome in EC-sensitized BALB/c mice, we wondered, to what extent the overall presence of intestinal and skin microbiome plays a role in the sensitization and severity of allergic response to EC OVA-sensitization and i.g. challenge in BALB/c mice. Compared to SPF mice, GF mice developed hypothermia but not diarrhea although they had higher mast cell numbers in jejunum, increased MCPT-1 production in jejunum and serum, and increased arachidonate 5-lipoxygenase (*alox5*) mRNA expression. In previous studies, we (13) and others (76) have shown that GF mice have negligible incidence of diarrhea, but all mice developed strong Th2 immune response after the i.p. OVA-sensitization and intragastric challenges. Similarly, here we determined increased levels of OVA-specific IL-4, IL-5, IL-10, IL-13 in splenocytes from GF compared to SPF OVA-treated mice but OVA-specific IgE and IgG1 levels were comparable in both OVA-treated groups (GF and SPF). These results are in contrast to the study by Stefka et al. (77), who demonstrated a significant increase in peanut-specific IgE and IgG1 levels in GF mice compared to SPF mice. It should be noted that they used a different mouse model of food allergy in which the mice were sensitized by intragastric gavages with antigen admixed with cholera toxin (CT). CT is known as a potent mucosal adjuvant which induces mobilization and maturation of immune cells in the gut (78) and induction of Th1/Th2/Th17 responses (79). Thus, administration of CT to GF mice could provide signals for the maturation or recruitment of MC to the gut tissue. Hong et al. (80) showed that food antigens itself can drive the spontaneous IgE upregulation in GF and antibiotics-treated mice. Disrupted intestinal microbiota by antibiotic treatment has been associated with more severe allergic phenotypes also in various other allergy models (76, 77, 81). Conversely, the re-colonization of GF mice by microbiota has been shown to dampen the allergic response, however the outcome depends on the complexity of microbiota and the age of mice at time of colonization (82). Thus, microbiota seems to influence the MCs accumulation in jejunum and food allergy symptoms, but not allergen sensitization in this model. Previously (13), we have shown that i.p. sensitized and OVA-challenged GF mice exhibited no symptoms of food allergy (diarrhea and anaphylactic hypothermia) due to impaired functionality and homing of MCs into the jejunum, accompanied by low levels of MCPT-1 both locally in the jejunal tissue and systemically in sera. Here, the EC sensitization of GF mice almost doubled the number of mast cells, led to higher production of MCPT-1 in jejunum and serum and increased mRNA expression of *alox5* after OVA i.g. treatment. Similarly, Rodriguez et al. (64) showed increased level of MCPT-1 in blood of GF mice sensitized using cholera-toxin and challenged with β-lactoglobulin. Together, these studies indicate that allergen sensitization using the damage of the skin (tape-stripping or calcipotriol) or intestine (cholera-toxin) barrier could induce strong maturation of mast cell compartment, their homing to the intestine and development of FA symptoms even in GF conditions, whereas the systemic (i.p.) sensitization in GF mice fails to do so despite the strong Th2 response.

In conclusion, our study emphasizes the importance of careful selection of mouse strain (BALB/c or C57BL/6) when conducting experiments involving mast cell and experimental allergy models. Besides the mouse genetic background, the route of allergen sensitization and microbiome can both affect the susceptibility to the development of food allergy following the natural sensitization pathway through the disrupted skin barrier. This is the first study to show that the absence of microbiome in GF mice exacerbates certain aspects of the allergic response, particularly hypothermia, while altering other symptoms, like diarrhea in this mouse model of allergy. The study underlines the critical role the gut microbiome plays in moderating the severity and nature of food allergy symptoms, as the lack of microbial diversity exacerbates immune dysregulation in the development of food allergy.

## Data Availability

The datasets presented in this study can be found in online repositories. The names of the repository/repositories and accession number(s) can be found below: https://www.ncbi.nlm.nih.gov/sra/PRJNA1145657, PRJNA1145657.
