# Supplementary material for: Genetic background and microbiome drive susceptibility to epicutaneous sensitization and food allergy in adjuvant-free mouse model
Source: Front Immunol. 2025 Jan 29;15:1509691. doi: 10.3389/fimmu.2024.1509691 (PMC11814220; doi:10.3389/fimmu.2024.1509691)
Supplement: Supplementary file 1 [file DataSheet1.pdf]

Supplementary Figures

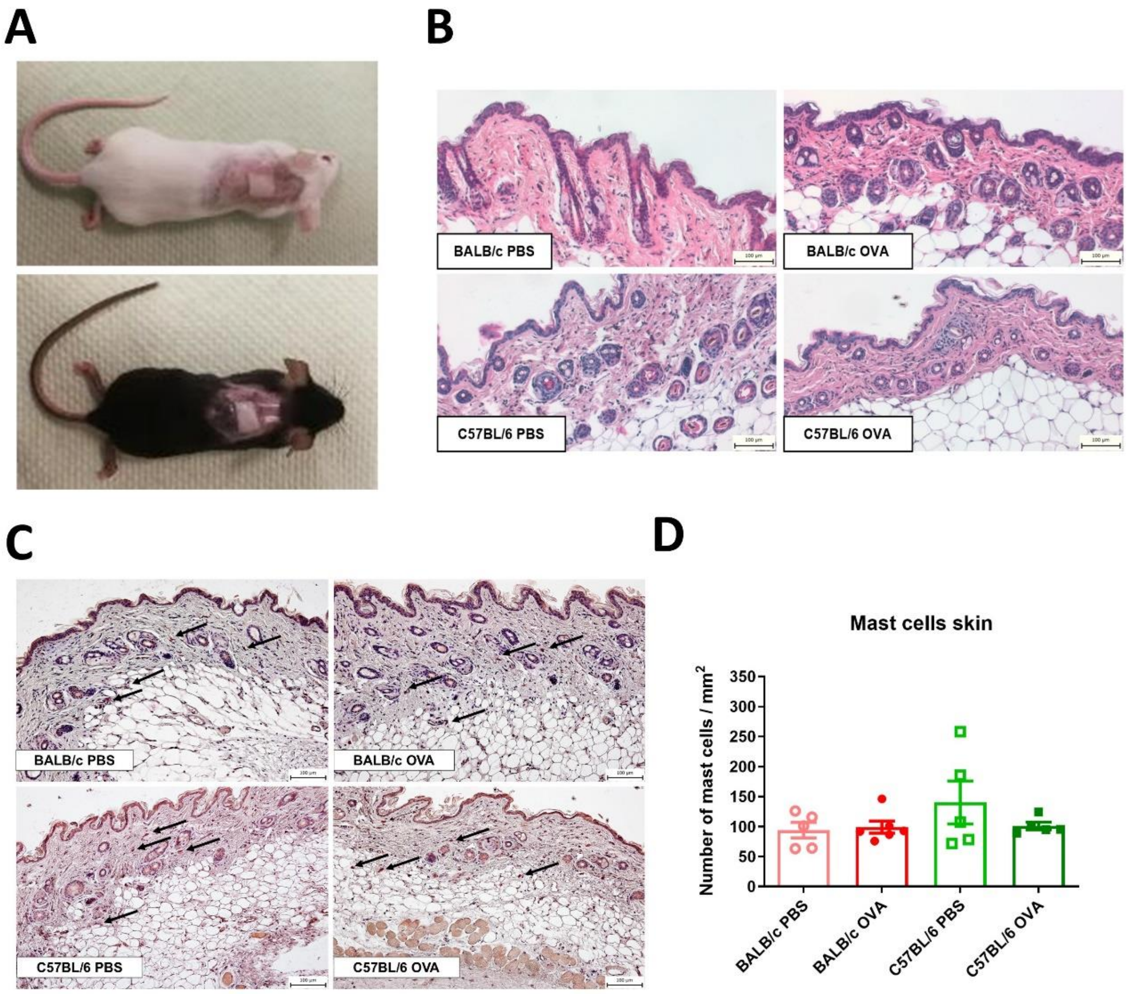

Figure S1: Impact of epicutaneous ovalbumin sensitization on skin histopathology (A) Representative picture of BALB/c and C57BL/6 OVA-treated mice. Altogether 50 µl of OVA (2mg/ml) was placed on a patch of sterile gauze and secured to the skin with a transparent bioocclusive dressing TegadermTM. (B) Histological staining of cutaneous section by hematoxylin/eosin (scale bars 100 µm). (C) Histological staining of mast cells by hematoxylin/pararosaniline in cutaneous section (scale bars 100 µm). (D) Quantification of mast cells per 1 mm^2 in cutaneous section (BALB/c PBS n = 5, BALB/c OVA n = 6, C57BL/6 PBS n = 5, C57BL/6 OVA n = 5 mice per group). Data are plotted as mean values ± SEM. One representative out of two independent experiments is shown. For statistical evaluation, one-way ANOVA with Tukey´s multiple comparison test was used.

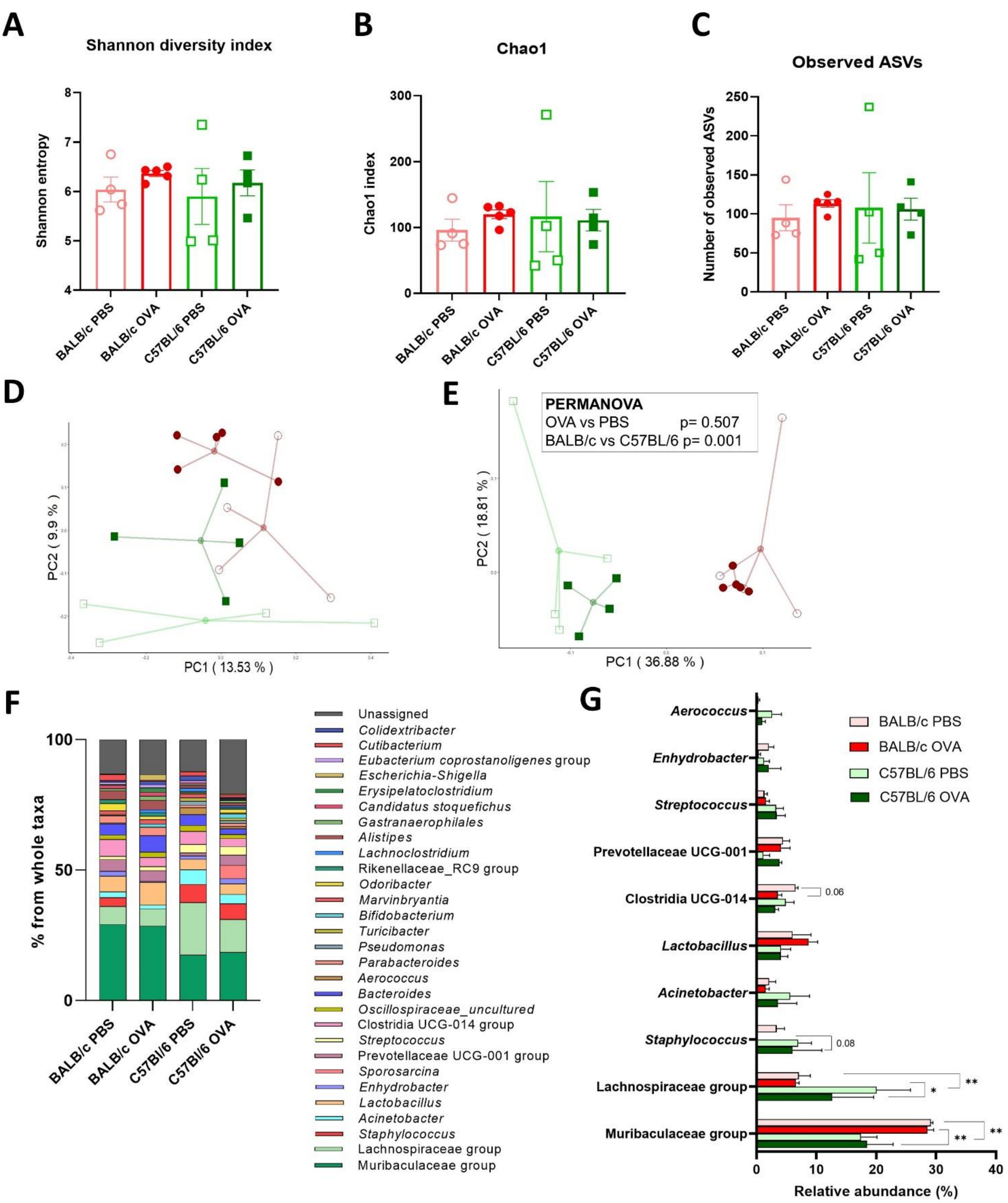

Figure S2: 16S rRNA sequencing analysis revealed the impact of mouse strain (BALB/c or C57BL/6) on distinct composition of skin bacteria. The α-diversity of skin microbiome represented by (A) Shannon entropy, (B) Chao1 index and (C) Observed ASVs. PCA plot showing β-diversity represented by the (D) unweighted and (E) weighted UniFrac distances among samples in cohorts. Points represent individual samples (BALB/c PBS - red empty circle, BALB/c OVA - red filled circle, C57BL/6 PBS - green empty square, C57BL/6 OVA – green filled square). PC1 and PC2 axes represent

the major variability. The PERMANOVA test was used for analysis of differences between treatment (OVA vs PBS) and mouse strains (BALB/c vs C57BL/6). (D) Taxonomy bar plot of bacterial genera was desighned for skin swab samples from experimental mice. Bars represent average abundance in percentage for all samples within a group. (E) Differences in relative abundances in selected highly abundant bacterial taxons among experimental groups. Significance between groups was determined by One-way ANOVA with Tukey´s multiple comparison test, *$p < 0.05$, **$p < 0.01$.

A

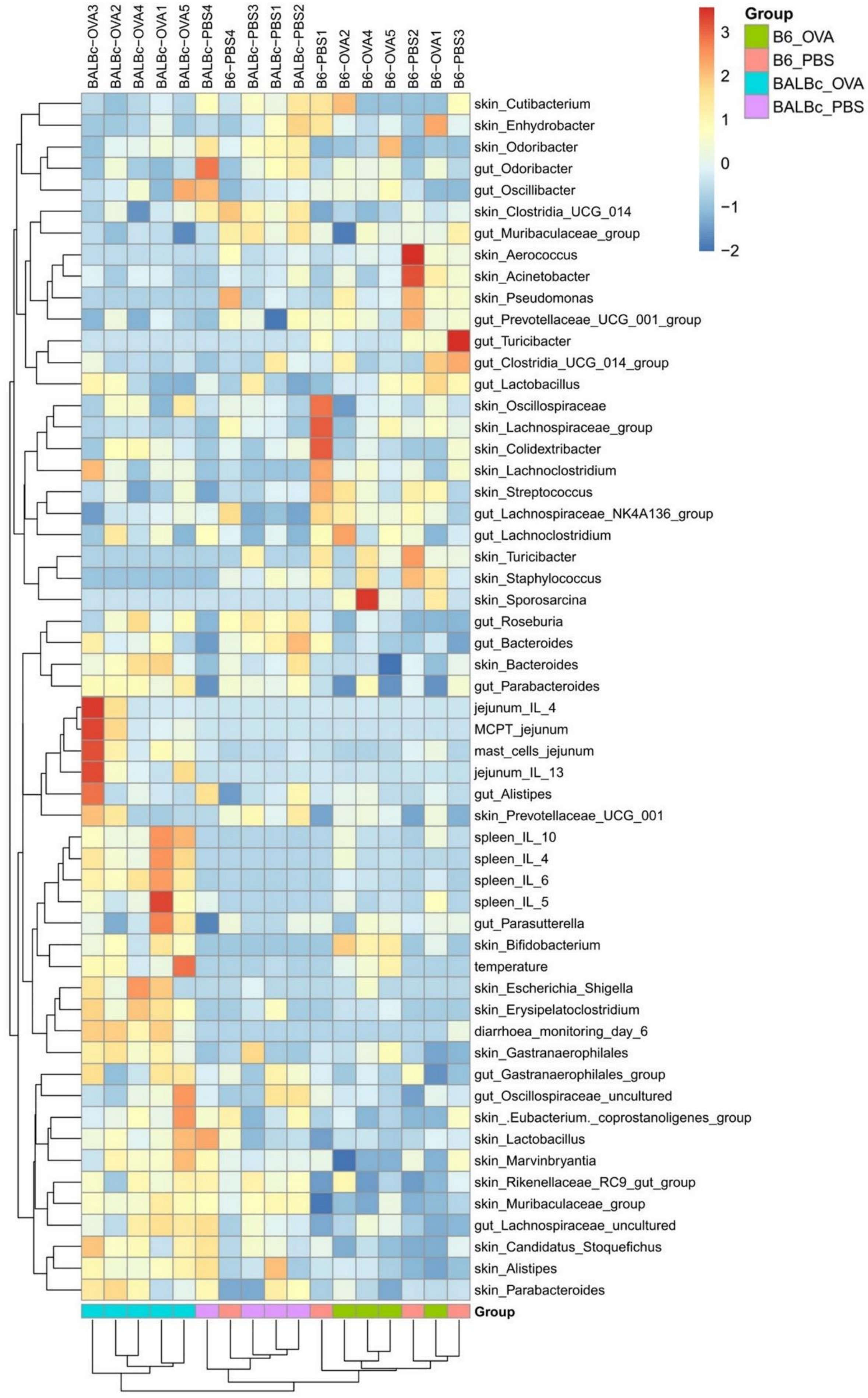
BALBc-OVA3
BALBc-OVA2
BALBc-OVA4
BALBc-OVA1
BALBc-OVA5
BALBc-PBS4
B6-PBS4
BALBc-PBS3
BALBc-PBS1
BALBc-PBS2
B6-PBS1
B6-OVA2
B6-OVA4
B6-OVA5
B6-PBS2
B6-OVA1
B6-PBS3
3
2
1
0
−1
−2
Group
B6_OVA
B6_PBS
BALBc_OVA
BALBc_PBS
skin_Cutibacterium
skin_Enhydrobacter
skin_Odoribacter
gut_Odoribacter
gut_Oscillibacter
skin_Clostridia_UCG_014
gut_Muribaculaceae_group
skin_Aerococcus
skin_Acinetobacter
skin_Pseudomonas
gut_Prevotellaceae_UCG_001_group
gut_Turicibacter
gut_Clostridia_UCG_014_group
gut_Lactobacillus
skin_Oscillospiraceae
skin_Lachnospiraceae_group
skin_Colidextribacter
skin_Lachnoclostridium
skin_Streptococcus
gut_Lachnospiraceae_NK4A136_group
gut_Lachnoclostridium
skin_Turicibacter
skin_Staphylococcus
skin_Sporosarcina
gut_Roseburia
gut_Bacteroides
skin_Bacteroides
gut_Parabacteroides
jejunum_IL_4
MCPT_jejunum
mast_cells_jejunum
jejunum_IL_13
gut_Alistipes
skin_Prevotellaceae_UCG_001
spleen_IL_10
spleen_IL_4
spleen_IL_6
spleen_IL_5
gut_Parasutterella
skin_Bifidobacterium
temperature
skin_Escherichia_Shigella
skin_Erysipelatoclostridium
diarrhoea_monitoring_day_6
skin_Gastranaerophilales
gut_Gastranaerophilales_group
gut_Oscillospiraceae_uncultured
skin_.Eubacterium._coprostanoligenes_group
skin_Lactobacillus
skin_Marvinbryantia
skin_Rikenellaceae_RC9_gut_group
skin_Muribaculaceae_group
gut_Lachnospiraceae_uncultured
skin_Candidatus_Stoquefichus
skin_Alistipes
skin_Parabacteroides
Group

B

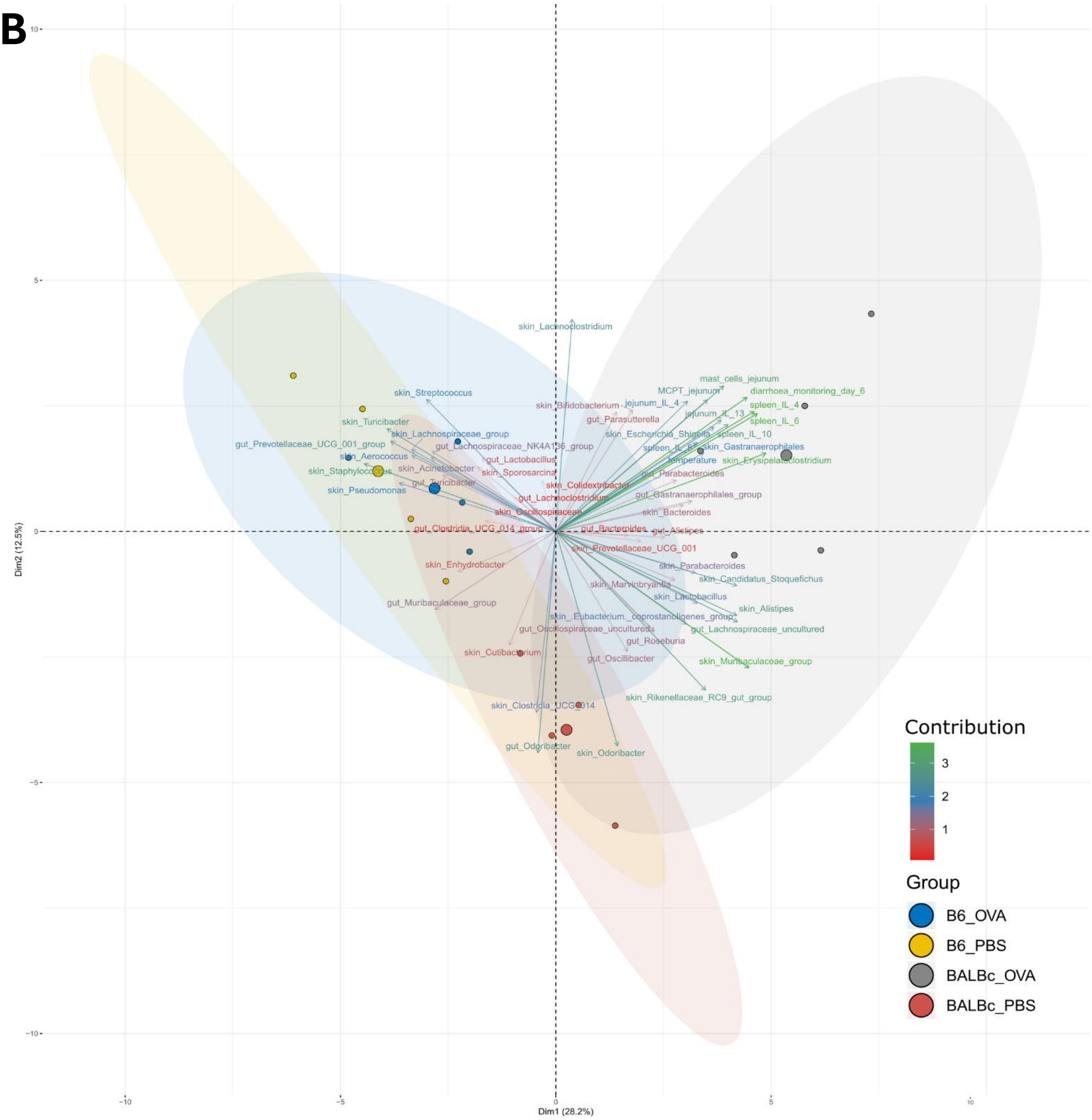

Figure S3: Multivariate analysis investigating the relationship between gut or skin microbiota abundance and the main features of immunological response to food allergen. Anaphylactic hypothermia and diarrhea, cytokine response in spleen (IL4, IL-5, IL-10 and IL-13), IL-4, IL-13, MCPT-1 cytokines and number of mast cells in jejunum and the abundance of the main bacterial taxa in the gut and on the skin of mice were subjected to the analysis. (A) Heatmap conclusively clusters the experimental and control groups of mice or the related immunological features and skin and gut bacterial taxa. (B) Principal component analysis (PCA) depicts the contribution of immunological factors or bacterial taxa to the clustering of BALB/c OVA group (BALBc_OVA) from C57BL/6 OVA (B6_OVA) and control groups (BALBc_PBS; B6_PBS). The most contributing factors are shown in green color, the least contributing factors are shown in red color. The data for immunological response and microbiome analysis from one representative experiment are included to the multivariate analysis.

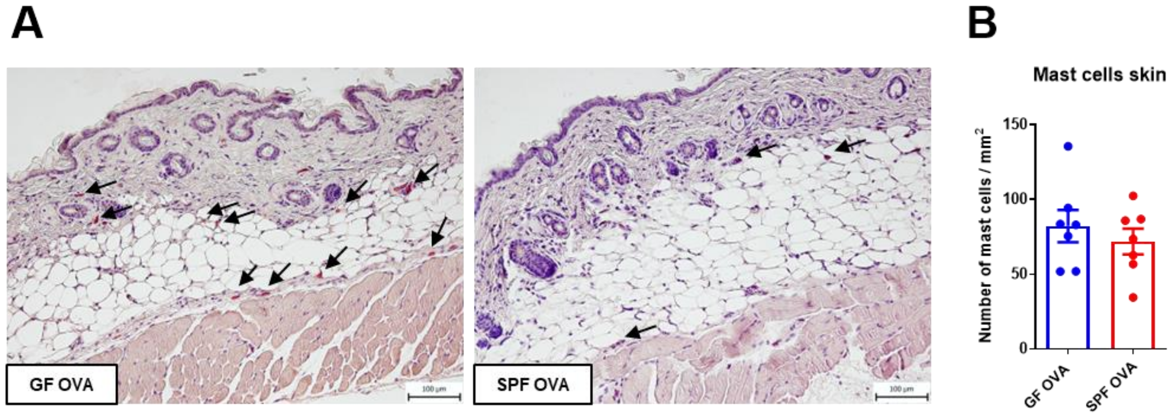

Figure S4: Impact of epicutaneous ovalbumin sensitization on the level of mast cells in the skin of germ-free mice (A) Histological staining of mast cells by hematoxylin/pararosaniline in cutaneous sections from germ-free (GF) and specific pathogen-free (SPF) BALB/c mice (scale bars 100 μm). (B) Quantification of mast cells per 1 mm^2 in cutaneous section (GF n = 7, SPF n = 7 mice per group). Unpaired t-test was used for comparison between experimental groups of mice.
