# Supplementary material for: Genetic background and microbiome drive susceptibility to epicutaneous sensitization and food allergy in adjuvant-free mouse model
Source: Front Immunol. 2025 Jan 29;15:1509691. doi: 10.3389/fimmu.2024.1509691 (PMC11814220; doi:10.3389/fimmu.2024.1509691)

Supplementary Material

Table S1. Sequences of real-time PCR primers

Gene	Forward primer	Reverse primer
tbp	5′-TGGTGTGCACAGGAGCCAAG-3′	5′- TTCACATCACAGCTCCCCAC-3′
alox5	5′-TCTTCCTGGCACGACTTTGCTG-3′	5′-GCAGCCATTCAGGAACTGGTAG-3′
hdc	5′-GATGGAGCCCTGTGAATACCG-3′	5′-CTGCACATTTGGAGTCACCTG-3′

Table S2: Rarefaction curves of 16S rRNA analysis for (A) fecal and (B) skin samples plotting sequencing depth and observed features.

A

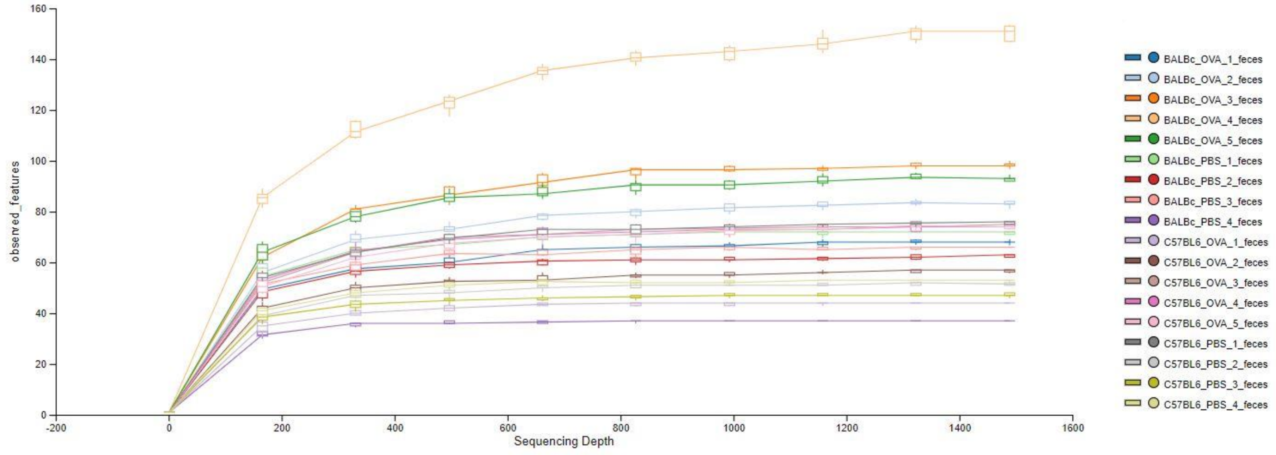

B

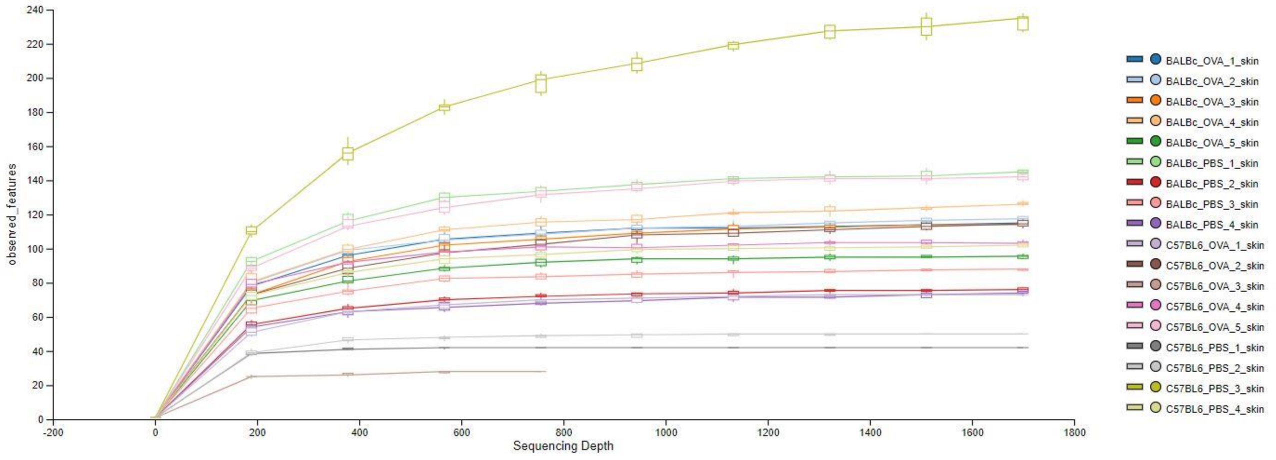

Supplement: Supplementary file 2 [file DataSheet2.pdf]
